# A novel role for the mineralocorticoid receptor in glucocorticoid driven vascular calcification

**DOI:** 10.1016/j.vph.2016.04.005

**Published:** 2016-11

**Authors:** Dongxing Zhu, Nabil A. Rashdan, Karen E. Chapman, Patrick WF Hadoke, Vicky E. MacRae

**Affiliations:** a The Roslin Institute and Royal (Dick) School of Veterinary Studies, University of Edinburgh, Midlothian EH25 9RG, UK; b University/BHF Centre for Cardiovascular Sciences, University of Edinburgh, The Queen's Medical Research Institute, 47 Little France Crescent, Edinburgh EH16 4TJ, UK

## Abstract

Vascular calcification, which is common in the elderly and in patients with atherosclerosis, diabetes and chronic renal disease, increases the risk of cardiovascular morbidity and mortality. It is a complex, active and highly regulated cellular process that resembles physiological bone formation. It has previously been established that pharmacological doses of glucocorticoids facilitate arterial calcification. However, the consequences for vascular calcification of endogenous glucocorticoid elevation have yet to be established. Glucocorticoids (cortisol, corticosterone) are released from the adrenal gland, but can also be generated within cells from 11-keto metabolites of glucocorticoids (cortisone, 11-dehydrocorticosterone [11-DHC]) by the enzyme, 11β-hydroxysteroid dehydrogenase type 1 (11β-HSD1). In the current study we hypothesized that endogenous glucocorticoids facilitate vascular smooth muscle cell (VSMC) calcification and investigated the receptor-mediated mechanism underpinning this process.

*In vitro* studies revealed increased phosphate-induced calcification in mouse VSMCs following treatment for 7 days with corticosterone (100 nM; 7.98 fold; *P* < 0.01), 11-DHC (100 nM; 7.14 fold; *P* < 0.05) and dexamethasone (10 nM; 7.16 fold; *P* < 0.05), a synthetic glucocorticoid used as a positive control. Inhibition of 11β-HSD isoenzymes by 10 μM carbenoxolone reduced the calcification induced by 11-DHC (0.37 fold compared to treatment with 11-DHC alone; *P* < 0.05). The glucocorticoid receptor (GR) antagonist mifepristone (10 μM) had no effect on VSMC calcification in response to corticosterone or 11-DHC. In contrast, the mineralocorticoid receptor (MR) antagonist eplerenone (10 μM) significantly decreased corticosterone- (0.81 fold compared to treatment with corticosterone alone; *P* < 0.01) and 11-DHC-driven (0.64 fold compared to treatment with 11-DHC alone; *P* < 0.01) VSMC calcification, suggesting this glucocorticoid effect is MR-driven and not GR-driven. Neither corticosterone nor 11-DHC altered the mRNA levels of the osteogenic markers *PiT-1*, *Osx* and *Bmp2*. However, DAPI staining of pyknotic nuclei and flow cytometry analysis of surface Annexin V expression showed that corticosterone induced apoptosis in VSMCs.

This study suggests that in mouse VSMCs, corticosterone acts through the MR to induce pro-calcification effects, and identifies 11β-HSD-inhibition as a novel potential treatment for vascular calcification.

## Graphical abstract

**Figure:**
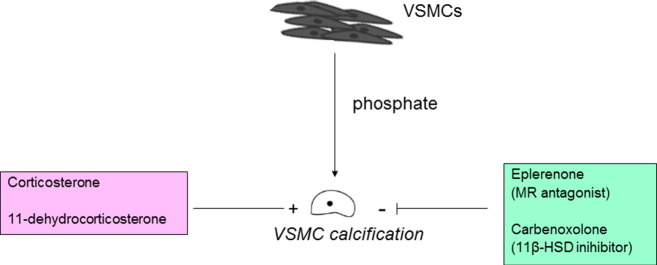

## 1 Introduction

Vascular calcification is a marker of increased cardiovascular disease risk in aging, including in diabetes, atherosclerosis and chronic kidney disease (CKD) [23], [50]. The etiology of mineral accumulation within the vasculature shares many similarities with that of bone formation. Indeed, several studies have reported that vascular smooth muscle cells (VSMCs), the predominant cell type involved in vascular calcification, can undergo phenotypic transition to osteoblastic, chondrocytic and osteocytic cells in a calcified environment [16], [49]. Furthermore, phosphate accelerates this trans-differentiation process, with the loss of characteristic smooth muscle markers and the increased expression of osteoblastic markers (e.g. Osterix, PiT-1 and BMP2) [28], [31], [51]. Vascular calcification can also proceed through mechanisms involving the reciprocal loss of recognized calcification inhibitors including inorganic pyrophosphate (PPi), fetuin A and Matrix Gla Protein [16], [25], [30], [36].

Physiological glucocorticoids — primarily cortisol in humans and corticosterone in rats and mice — are steroid hormones produced by the adrenal cortex. Local glucocorticoid action on target tissues is determined by intracellular metabolism by the two isozymes of 11β-hydroxysteroid dehydrogenase (11β-HSD) which catalyze interconversion of active cortisol and corticosterone with inert cortisone and 11-dehydrocorticosterone [11]. 11β-HSD type 1, a predominant reductase in most intact cells, catalyzes the regeneration of active glucocorticoids, thus amplifying cellular action. 11β-HSD2 is a high-affinity dehydrogenase that inactivates glucocorticoids [4]. Both isozymes of 11HSD are modestly expressed in the blood vessel wall, suggesting that they can influence vascular function by regulating local availability of active glucocorticoids [11].

In the absence of 11β-HSD2, endogenous glucocorticoids can bind to the mineralocorticoid receptor (MR) as well as the glucocorticoid receptor (GR) [37]. Both MR and GR belong to the same nuclear hormone receptor superfamily, and share high sequence identity. MR has higher affinity for glucocorticoids than GR, and both receptors are expressed in the cells of the vasculature [10]. Glucocorticoids can activate the MR in VSMCs (11β-HSD2 is not expressed here), inducing pathways that are central to cell proliferation and differentiation [27].

Glucocorticoids are frequently permissive, co-operative or synergistic [48]. Indeed a permissive role of glucocorticoids in triggering cell transdifferentiation has previously been established in the conversion of pancreatic cells into hepatocytes [38]. Furthermore, glucocorticoids exert complex actions on calcium mobilisation and bone metabolism, regulating bone resorption and formation [13], intestinal calcium absorption and renal calcium excretion [8]. Therefore it is essential to establish the consequences for vascular calcification of endogenous glucocorticoid elevation given the high circulating calcium levels commonly observed in patients with this pathology [2].

Dexamethasone, a potent synthetic glucocorticoid which is primarily active at the GR, induces an osteoblastic differentiation pathway in many different mesenchymal-derived cell types *in vitro* [1], [5], [47], including VSMCs [29], [39]. Whilst the established pro-calcification actions of dexamethasone on VSMCs [18], [29] are presumed to be mediated *via* GR, plausibly endogenous corticosteroids may modulate VSMC calcification *via* MR. This is therapeutically important to ascertain, as vascular calcification is independently correlated with adverse cardiac events [50], and MR antagonism is highly successful in reducing mortality in heart failure [45]; aldosterone antagonists such as spironolactone and eplerenone have been shown to improve cardiovascular outcomes and prevent ischaemic events in cardiovascular patients [32], [33]. Corticosterone has been shown to induce rapid MR signaling in VSMCs that involves mitogen-activated protein kinase kinase (MEK)/extracellular signal-regulated kinase (ERK)-dependent pathways, suggesting that glucocorticoids may contribute to vascular disease *via* MR receptor signaling [27]. Recent studies have shown that aldosterone-induced activation of MR promotes osteoblastic differentiation and calcification of VSMCs [15] through a mechanism involving the stimulation of spironolactone-sensitive, PiT-1 dependent signaling [46].

An additional level of control over endogenous corticosteroid action is provided by the HSD isoenzymes, whose role in vascular calcification has yet to be elucidated. The induction of local glucocorticoid generation through increased 11β-HSD1 expression (> 10 fold) and activity (> 4 fold) by inflammatory cytokines and glucocorticoids is well documented in fibroblasts and osteoblasts [19], [43], which both have the capacity to calcify [3], [42].

It is therefore essential to establish the consequences for vascular calcification of endogenous glucocorticoid elevation and potential strategies for inhibition of calcification. The aims of this study were to undertake *in vitro* murine VSMC calcification studies to investigate both the identity of the receptor and the role of the 11β-HSD isoenzymes in corticosterone-induced calcification.

## 2 Materials and methods

### 2.1 Mice

All animal experiments were performed under UK Home Office licensed approval in accordance with Directive 2010/63/EU of the European Parliament and were maintained in accordance with Home Office guidelines for the care and use of laboratory animals. C57BL/6 mice were supplied by Charles River Laboratories (Harlow, Essex, UK).

### 2.2 Preparation of VSMCs

Mice were euthanized by cervical dislocation. Primary murine VSMCs were isolated as described [24]. Briefly, after removal of the adventitia, the aorta was opened to expose the endothelial layer under a dissection microscope. Tissues from eight animals were pooled and incubated with 1 mg ml^− 1^ trypsin (Invitrogen, Paisley, UK) for 10 min in order to enable the removal of any remaining adventitia and endothelium through further dissection. Following overnight incubation at 37 °C in a humidified atmosphere of 95% air/5% CO_2_ in “growth medium” (α-MEM supplemented with 10% fetal bovine serum and 1% gentamicin, all from Invitrogen), tissues were digested with 425 U/ml collagenase type II (Worthington Biochemical Corporation, Lakewood, USA) for 5 h. Cell suspensions were centrifuged at 2000 *g* for 5 min. The cell pellet was washed and resuspended in growth medium. Isolated VSMCs were passaged in growth medium twice in T25 tissue culture flasks (Greiner Bio-one, GmbH, Frickenhausen, Baden-Wurttemberg, Germany) coated with 0.25 μg/cm^2^ laminin (Sigma, Poole, UK) to promote maintenance of the contractile differentiation state [17]. VSMCs were subsequently seeded at a density of 1.5 × 10^4^/cm^2^ in 12-well plates.

### 2.3 Induction of VSMC calcification

*In vitro* calcification of VSMCs was induced by culturing cells in growth medium containing 3 mM inorganic phosphate (a mixture of NaH_2_PO_4_ and Na_2_HPO_4_, pH 7.4, Sigma) for up to 14 days, with a medium change every 3 days, as previously described [53]. The effects of glucocorticoids in FBS were assessed through comparison of charcoal-stripped and standard FBS (Life Technologies Ltd). Cells were treated with corticosterone (1–100 nM) (Sigma), 11-DHC (1–100 nM) (Steraloids, Newport, USA), carbenoxolone (10 μM) (Sigma), dexamethasone (1–100 nM) (Sigma), mifepristone (10 μM) (Sigma) or eplerenone (10 μM) (Sigma). The *in vitro* levels of corticosterone and 11-DHC used in the present study reflect those found *in vivo*. Plasma corticosterone levels in mice range from 20 nM (nadir, morning) to ~ 150 nM (peak, evening) and stress levels are typically 200–250 nM. Basal levels of plasma 11-DHC in mice have been reported at 2–5 nM and stress levels > 30 nM [12].

### 2.4 Determination of VSMC calcification

Calcium deposition was quantified by HCl leaching, as described previously [52]. Briefly, cells were washed twice with phosphate buffered saline (PBS) and incubated with 0.6 M HCl at room temperature for 24 h. Calcium content was determined colorometrically by a stable interaction with phenolsulphonethalein using a commercially available kit (Randox Laboratories Ltd., County Antrim, UK), corrected for total protein concentration (Bio-Rad Laboratories Ltd., Hemel Hempstead, UK), and presented as a fold change compared with control. Calcium deposition was also evaluated by alizarin red staining. Cells were washed twice with PBS, fixed in 4% paraformaldehyde for 5 min at 4 °C, stained with 2% alizarin red (pH 4.2) for 5 min at room temperature and rinsed with distilled water.

### 2.5 Analysis of gene expression

VSMCs were treated with corticosterone (Sigma) or 11-DHC (Steraloids, Newport, USA), for 48 h in serum free α-MEM (Invitrogen). RNA was extracted using RNeasy total RNA (Qiagen Ltd., Crawley, West Sussex, UK), according to the manufacturer's instructions. RNA was quantified and reverse transcribed as previously described [26]. Levels of specific mRNAs were measured using the SYBR green detection method (Roche, East Sussex, UK) as previously reported [42]. Primers were obtained from Qiagen (sequences not disclosed) for *PiT-1* (NM_001159593), *Bmp2* (NM_007553) and *Msx2* (NM_013601).

### 2.6 Quantification of apoptosis

On reaching confluence, cells were serum starved for 24 h, then treated with 100 nM corticosterone for 48 h. Cells were harvested by trypsinization and re-suspended in 25 μl 1% trypan blue (diluted 50% in PBS). Live cells, which exclude trypan blue and dead cells (stained blue) were counted using a hemocytometer, and the results expressed as the percentage of cells that were dead. Apoptotic VSMCs were determined by manually counting pyknotic nuclei after staining with DAPI (Invitrogen) as previously described [9]. Additionally, cells in different stages of apoptosis were analyzed by flow cytometry using the TACS Annexin-V-FITC apoptosis detection kit (R&D systems, Abingdon, UK), according to the manufacturer's instructions. Non-apoptotic cells do not stain with either Annexin-V FITC or propidium iodide. Early apoptotic cells are stained with Annexin-V FITC but not propidium iodide (green fluorescence). Late apoptotic cells are stained with both Annexin-V FITC and propidium iodide (dual green and red fluorescence). Necrotic cells are only stained with propidium iodide (red fluorescence). 10,000 cell events were recorded on a BD FACS Calibur and data were analyzed with FlowJo 8.8.4 flow cytometry analysis software (Tree Star Inc., Ashland, Oregon, USA).

### 2.7 Statistical analysis

General Linear Model analysis and the Students t-test were used to assess the data. All data are expressed as the mean ± S.E.M. Statistical analysis was performed using Minitab 16. P < 0.05 was considered to be significant.

## 3 Results

### 3.1 Glucocorticoids facilitates VSMC calcification

**Fig. 1:**
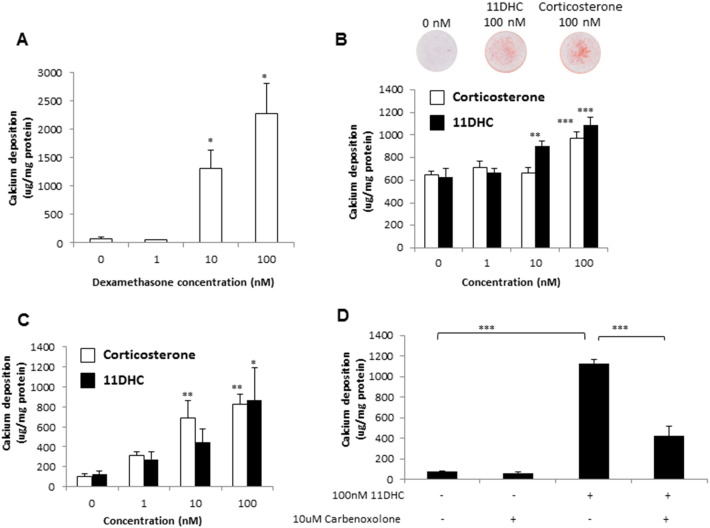
Dexamethasone, corticosterone and 11-DHC all glucocorticoids induce VSMC calcification. Effect of (A) dexamethasone (1–100 nM), (B, C) corticosterone (white bar) and 11-dehydrocorticosterone (11DHC; filled bar) (1–100 nM) in the presence of standard and charcoal-stripped fetal bovine serum (FBS) respectively and (D) carbenoxolone (10 μM) in the presence or absence of 11DHC (100 nM) on calcium deposition in VSMCs cultured in high phosphate (Pi) (3 mM Pi) for 7 days, as determined by alizarin red staining and/or quantitative HCL leaching (μg/mg protein) (*n* = 6). Results are presented as mean ± S.E.M. *P < 0.05; **P < 0.01; ***P < 0.001 compared with corresponding 0 nM treatment.

We initially examined the effects of physiological glucocorticoids on the calcification of VSMCs, with the synthetic glucocorticoid dexamethasone used as a positive control. Since arterial calcification is highly correlated with elevated serum Pi levels, VSMCs were cultured in growth medium containing high (3 mM) Pi as previously described ([53]; Zhu et al., 2016). Cells were treated with dexamethasone, corticosterone or 11-DHC (1–100 nM) for up to 7 days. Dexamethasone treatment significantly increased calcium deposition of VSMCs (17.16 fold, *P* < 0.05, Fig. 1A). Both corticosterone and 11-DHC significantly increased calcium deposition in VSMCs cultured with un-stripped FBS (1.51 fold, *P* < 0.001 and 1.72 fold respectively, *P* < 0.001; Fig. 1B). Surprisingly, 11-DHC was more potent than corticosterone, with a significant effect at 10 nM, compared to 100 nM for corticosterone. Using FBS stripped of steroids markedly increased the magnitude of the effects of both corticosterone and 11-DHC on VSMC calcification (7.98 fold, *P* < 0.01 and 7.14 fold, *P* < 0.05 respectively; Fig. 1C). The 11β-HSD inhibitor carbenoxolone notably reduced 11-DHC-induced calcification of VSMCS (0.37 fold compared to treatment with 11-DHC alone, *P* < 0.001, Fig. 1D), confirming the 11β-HSD isoenzymes as key regulators of intracellular glucocorticoid levels [4].

### 3.2 Corticosterone and 11-DHC facilitate VSMC calcification through the MR

**Fig. 2:**
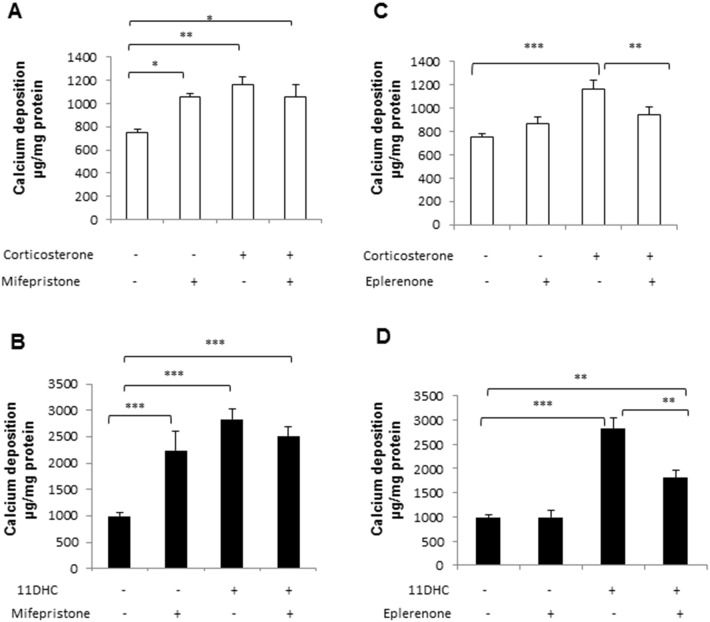
Endogenous glucocorticoids induce VSMC calcification through the mineralocorticoid receptor (MR). Effect of (A) mifepristone (10 μM) and (B) eplerenone (10 μM) in the presence of corticosterone (100 nM) and (C) mifepristone (10 μM) and (D) eplerenone (10 μM) in the presence of 11-dehydrocorticosterone (11HDC; 100 nM) on calcium deposition in VSMCs cultured in high phosphate (Pi) (3 mM Pi) for 7 days, as determined by quantitative HCL leaching (μg/mg protein) (*n* = 6). Results are presented as mean ± S.E.M. *P < 0.05; **P < 0.01; ***P < 0.001.

In order to establish whether corticosterone acts *via* GR or MR to induce VSMC calcification, antagonists of both MR (eplerenone) and GR (mifepristone) were employed. Mifepristone (10 μM) itself potentiated VSMC calcification, but did not alter the response to corticosterone (Fig. 2A) or 11-DHC (Fig. 2B). However, eplerenone (10 μM) significantly attenuated the effect of corticosterone (0.81 fold compared to treatment with corticosterone alone; *P* < 0.01) and 11-DHC (0.64 fold compared to treatment with 11-DHC alone; *P* < 0.01) on VSMC calcification.

### 3.3 Corticosterone and 11-DHC induce VSMC apoptosis

**Fig. 3:**
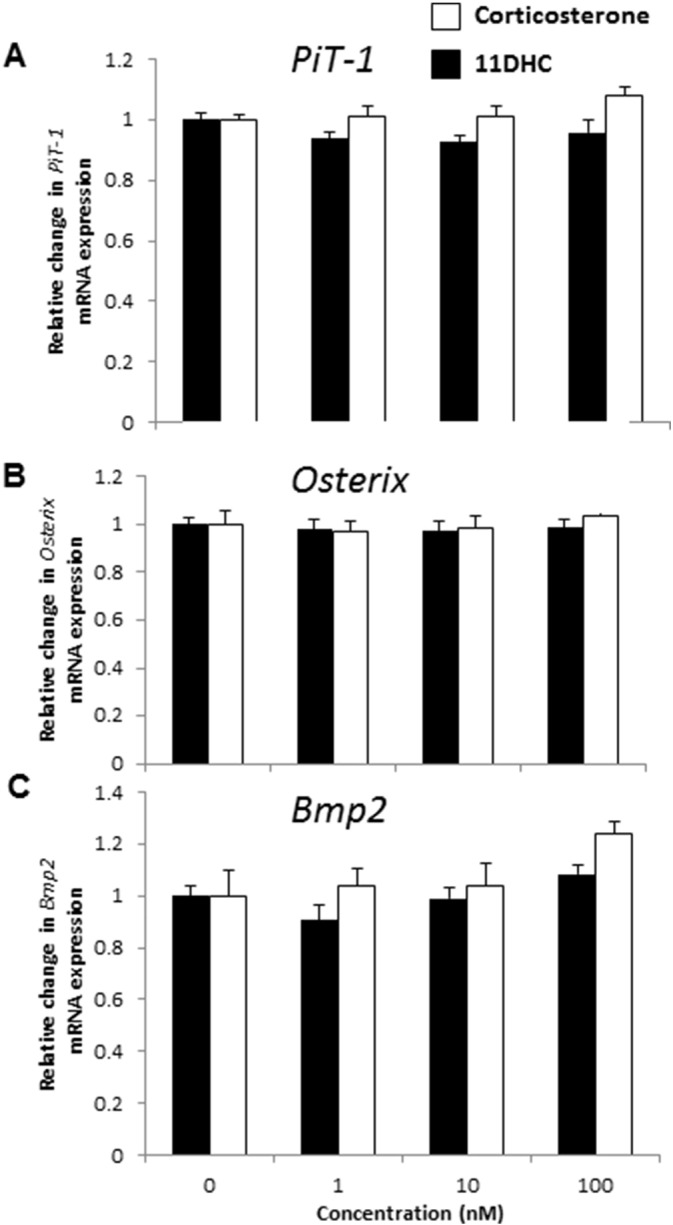
Endogenous glucocorticoids do not induce osteogenic marker expression in VSMCs. Fold change in the mRNA expression of osteogenic markers (A) *PiT-1*, (B) *Osx*, and (C) *Bmp2* (*n* = 4) following treatment with corticosterone (white bar) and 11-dehydrocorticosterone (11DHC; filled bar) (1–100 nM). 0 nM treatment expressed as 1 to indicate fold change for each gene of interest. Results are presented as mean ± S.E.M. *P < 0.05; **P < 0.01; ***P < 0.001 compared with corresponding 0 nM treatment.

**Fig. 4:**
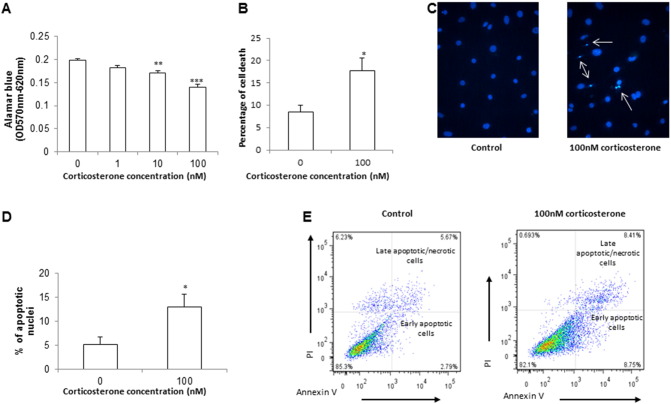
Endogenous glucocorticoids induce apoptosis in VSMCs. Effect of corticosterone (1–100 nM) on (A) Alamar blue uptake (OD 570 nm–620 nm), (B) trypan blue uptake (%), (C and D) apoptotic nuclei as determined by DAPI staining (%) and (E) Annexin V staining of isolated cells as assessed by FACS analysis. Results are presented as mean ± S.E.M. *P < 0.05; **P < 0.01; ***P < 0.001 compared with corresponding 0 nM treatment.

Published data implicate the transdifferentiation of VSMCs to an osteoblast-like phenotype as a mechanism underlying the effects of dexamethasone upon vascular calcification [29]. We therefore next tested whether the same is true for physiological glucocorticoids. Interestingly, neither corticosterone nor 11-DHC altered mRNA levels of key osteogenic markers: *PiT-1* (Fig. 3A), *Osx* (Fig. 3B) and *Bmp2* (Fig. 3C). Recent reports have highlighted apoptosis as essential for the initiation and progression of phosphate-induced vascular calcification [41]. Therefore we undertook a detailed assessment of apoptosis following glucocorticoid treatment. Corticosterone treatment significantly reduced cell viability (2.07 fold; *P* < 0.01; Fig. 4A), and conversely increased cell death (2.53 fold; *P* < 0.05; Fig. 4B) and apoptosis (Fig. 4C–E) as determined by DAPI staining of pykrotic nuclei and FACS analysis of cells positively stained for Annexin V.

## 4 Discussion

It is well established that glucocorticoids mediate changes in vascular growth, function and structure [10]. Previous studies in VSMCs have reported the pro-calcification effects of dexamethasone, a potent synthetic glucocorticoid. Here we demonstrate for the first time the facilitation of vascular calcification by both the active physiological glucocorticoid corticosterone and the inactive metabolite 11-DHC.

Currently 11β-HSD inhibition is therapeutically employed in topical preparations for the management of mouth ulcers [14]. Furthermore, 11β-HSD-inhibition has been shown to correct insulin resistance in rodent models of CKD, a hallmark of this disease in patients being vascular calcification [2]. The *in vitro* experiments undertaken in this study therefore identify 11β-HSD inhibition as a plausible treatment for vascular calcification, and requires further interrogation *in vivo*.

Whilst VSMC calcification is facilitated by GR selective dexamethasone (Fig. 1; [29]), and macrophage-specific GR inactivation reduces vascular calcification in a mouse model of atherosclerosis [34], the pro-calcification effects of corticosterone and 11-DHC do not appear to be mediated through GR signaling. Furthermore, whilst dexamethasone has been previously reported to accelerate the osteogenic differentiation of vascular pericytes [18] and bovine VSMCs [29], in the present study *Osterix*, *Bmp2* and *PiT-1* expression were unaltered in VSMCs treated with corticosterone or 11DHC. Interestingly, these data contrast with a previous demonstration of up-regulated *PiT-1* in response to MR signaling [46]. This paradigm likely reflects different mechanisms underpinning the calcification permitted by synthetic and physiological glucocorticoids. Indeed, our studies suggest that the pro-calcification effects of corticosterone and 11-DHC are mediated directly through MR, corroborating previous work in VSMCs showing the activation of MR signaling by glucocorticoids [27]. Furthermore, our data complement previous work revealing that CVCs (calcifying vascular cells; subpopulations of VSMCs which have been found to spontaneously calcify *in vitro*) contain MRs which function as transcriptional regulators that can be activated by both aldosterone and cortisol [15]. Indeed aldosterone administration has been shown to directly stimulate CVC calcification, an effect abolished by aldosterone antagonism with spironolactone [15]. Moreover, vascular calcification facilitated by hyperaldosteronism due to klotho deficiency has also been shown to be mitigated by spironolactone treatment in mice [46]. Further studies are therefore required to directly compare the effects of aldosterone treatment on VSMC calcification with that of corticosterone and 11-DHC.

The present study also highlights that the effect of corticosterone and 11-DHC on driving VSMC calcification is more pronounced when charcoal stripped media is used to remove endogenous steroid ligands, suggesting an activating effect of endogenous MR ligands in normal, unstripped serum. These data support comparable findings demonstrating that aldosterone-induced CVC calcification *via* MR activation is also enhanced using charcoal stripped serum [15].

Mechanistically, our data reveal that corticosterone reduces cell viability and stimulates VSMC apoptosis. This process is essential for the initiation and progression of phosphate-induced vascular calcification [41], with apoptotic bodies exposing phosphatidylserine on the outer membranes, generating a potential calcium-binding site suitable for hydroxyapatite deposition [35], [40]. These results support previous reports demonstrating that glucocorticoids inhibit VSMC proliferation [22], [44] and induce apoptosis in a range of cell types including neuronal cells, growth plate chondrocytes and thymocytes [6], [7], [20], [21].

In conclusion, we have undertaken *in vitro* murine VSMC studies to provide new insights into the role of physiological glucocorticoids in vascular calcification. Our study suggests that corticosterone acts through the MR to induce pro-calcification effects. This data may have important health ramifications for patients receiving MR blockers. The previously established clinical cardiovascular benefits of eplerenone administration [33], in conjunction with our *in vitro* findings may pave the way for pre-clinical and clinical trials for the treatment of vascular calcification with eplerenone therapy. Inhibiting 11β-HSD isoenzymes and subsequently diminishing vascular calcification may also represent a novel potential pharmaceutical target for clinical intervention.
